# Hypertension predicts a poor prognosis in patients with esophageal squamous cell carcinoma

**DOI:** 10.18632/oncotarget.23774

**Published:** 2018-01-01

**Authors:** Jie Liang, Guodong Li, Jun Xu, Tong Wang, Yanyan Jia, Qinghua Zhai, Lihua Qiao, Miao Chen, Yajing Guo, Shujun Zhang

**Affiliations:** ^1^ Department of Medical Records and Statistics, Shanxi Provincial Cancer Hospital, Taiyuan, Shanxi, China; ^2^ Department of Hospital Administration, Shanxi Provincial Cancer Hospital, Taiyuan, Shanxi, China; ^3^ Department of General Surgery, Shanxi Provincial Cancer Hospital, Taiyuan, Shanxi, China; ^4^ Department of Medical Records and Follow-up, Shanxi Provincial Cancer Hospital, Taiyuan, Shanxi, China; ^5^ Department of Health Statistics, School of Public Health, Shanxi Medical University, Taiyuan, Shanxi, China

**Keywords:** ESCC, hypertension, hypoxia, VEGF, esophagectomy

## Abstract

**Background:**

We investigated the relationship between the preoperative hypertension and prognosis of esophageal squamous cell cancer (ESCC) patients who had underwent esophagectomy.

**Results:**

We detected 52% patients with hypertension, including 317 patients with newly diagnosed hypertension and 194 patients with history of hypertension. Compared with patients of normal blood pressure, all patients with hypertension and newly diagnosed hypertension were observed to have worse overall and ESCC-specific survival outcome (*p* < 0.05). After adjusted in multivariate Cox regression analysis, hypertension (HR: 1.343, 95% CI: 1.064, 1.695; HR: 1.315, 95% CI: 1.039, 1.664) and newly diagnosed hypertension (HR: 1.414, 95% CI: 1.095, 1.826; HR: 1.420, 95% CI: 1.098, 1.836) were inversely associated with overall and ESCC-specific survival outcome, respectively. While no association was found between history of hypertension and overall or ESCC-specific survival outcome (HR: 1.229, 95% CI: 0.892, 1.694; HR: 1.132, 95% CI: 0.812, 1.578).

**Conclusions:**

Hypertension was an independent risk factor and resulted in inferior prognosis for ESCC patients who had underwent esophagectomy.

**Methods:**

A total of 982 ESCC patients who had underwent esophagectomy from August 2010 to December 2015 were enrolled in our study with a follow up of 6 years. The Kaplan-Meier method and log-rank test were respectively used to calculate and compare survival rate, and Cox proportional hazards regression model was applied to identify independent prognostic factors.

## INTRODUCTION

Esophageal cancer (EC) with higher incidence and mortality has been still an aggressive malignant tumors worldwide [1]. EC is one of the highest prevalence tumors and the fourth leading cause of cancer-related death in China, and its age-standardized 5-year survival rate was only 20.9% [2]. The esophageal squamous cell carcinoma (ESCC) is the predominant histological type and accounts for over 90% of EC in China [1, 3]. Up until now, surgical resection has remained the mainstream treatment for ESCC. However, the long-term outcomes after esophagectomy are still not ideal, with the average 5-year survival rates at 20–30% [4, 5]. Therefore, it is necessary to detect the risk factors associated to prognosis of ESCC patients after surgical resection and further adopting adjunctive therapy to make it control.

Hypertension, a chronic disease, is among other acknowledged key risk factors for stroke and coronary heart disease. As reported, approximately 40% of adults above 25 years old had been diagnosed with hypertension in worldwide [6]. The influence of hypertension has been a field of investigation for long-time. Many studies have separately reported hypertension as an important risk factor for elevated cancer incidence and mortality. A combined analysis of the Nurses’ Health Study (NHS) and the Health Professionals Follow-up Study (HPFS) cohorts in the United States have examined hypertension as an independent risk factor of renal cell carcinoma incidence in a prospective study [7]. Other researchers identified that hypertension is associated with incidence and progression of kidney cancer [8, 9]. In addition, the results from other large prospective studies suggested that elevated blood pressure (BP) might be associated with an increased risk of cancer incidence, including fatal malignant melanoma [10, 11], pancreatic [12], endometrial [13], brain and bladder cancer [14, 15]. Observational studies have shown results for the relationship between hypertension and higher risk of cancer mortality. A meta-analysis based on 10 longitudinal studies of in total 47119 participants, hypertension is related to a 23% increased risk of cancer mortality [16]. The positive risk has been found that hypertension is associated with an increased risk of death from prostate cancer [17–20]. In a historical cohort study of women diagnosed with breast cancer, hypertension is associated with total and breast cancer-specific mortality [21]. Another study including 17498 participants showed that BP is inversely associated with mortality from leukemia and pancreatic cancer but positively associated with mortality attributed to liver and rectal cancer [10]. Since hypertension is a risk factor of cancer incidence and mortality, we supposed whether this trend could be found in hypertension and prognosis of cancer. However, fewer of studies have identified the relationship between hypertension and prognosis of cancer directly. This relationship is more often to be explored indirectly by taking hypertension as a trait of metabolic syndrome, which has been identified negatively associated with prognosis of cancer, such as breast cancer [22] and renal cell carcinoma [23]. Therefore, this study was designed to investigate the association between preoperative hypertension and prognosis among patients with ESCC.

## RESULTS

### Patients baseline characteristics and survival outcome

In the current study, the overall prevalence of hypertension in 982 patients was 52.0%. Among them, 317 were newly diagnosed hypertension and 194 had history of hypertension including 34.5% well-controlled and 65.5% poorly-controlled hypertension. Distributions of baseline characteristics based on different hypertension status were shown in Table 1. Patients with history of hypertension were significantly more likely to be older than patients with newly diagnosed hypertension and normal BP.

**Table 1:** Distributions of baseline characteristics based on different hypertension status among patients with ESCC

Variables	Without HBP (*n* = 471)	Newly diagnostic hypertension (*n* = 317)	History of hypertension			
			Well-controlled (*n* = 67)	Poorly-controlled (*n* = 127)	χ^2^	*p*
**Age (years)**					14.698	0.002
<63	224 (47.56)	138 (43.53)	20 (29.85)^*^	41 (32.28)^*^		
≥63	247 (52.44)	179 (56.47)	47 (70.15)	96 (67.72)		
**Gender**					0.626	0.890
Male	299 (63.48)	194 (61.20)	41 (61.19)	77 (60.63)		
Female	172 (36.52)	123 (38.80)	26 (38.81)	50 (39.37)		
**Family history**					1.881	0.597
No	387 (82.17)	255 (80.44)	51 (76.12)	100 (78.74)		
Yes	84 (17.83)	62 (19.56)	16 (23.88)	27 (21.26)		
**Smoking**					3.975	0.264
No	213 (45.22)	148 (46.69)	32 (47.76)	70 (55.12)		
Yes	258 (54.78)	169 (53.31)	35 (52.24)	57 (44.88)		
**Drinking**					0.957	0.812
No	324 (68.79)	222 (70.03)	43 (64.18)	86 (67.72)		
Yes	147 (31.21)	95 (29.97)	24 (35.82)	41 (32.28)		
**Depth of tumor infiltration**					15.810	0.200
Tis	6 (1.27)	5 (1.58)	0	0		
T1	77 (16.35)	56 (17.67)	16 (23.88)	29 (22.83)		
T2	90 (19.11)	56 (17.67)	16 (23.88)	28 (22.05)		
T3	287 (60.93)	188 (59.31)	34 (50.75)	69 (54.33)		
T4	11 (2.34)	12 (3.79)	1 (1.49)	1 (4.00)		
**Lymph node metastases**					5.629	0.131
N0	306 (64.97)	219 (69.09)	44 (65.67)	96 (75.59)		
N1–N3	165 (35.03)	98 (30.91)	23 (34.33)	31 (24.41)		
**Diameter (cm)**					0.734	0.865
<4.0	267 (56.69)	179 (56.47)	38 (56.72)	77 (60.63)		
≥4.0	204 (43.31)	138 (43.53)	29 (43.28)	50 (39.37)		
**Vascular infiltration**					2.990	0.393
No	371 (78.77)	249 (78.55)	55 (82.09)	108 (85.04)		
Yes	100 (21.23)	68 (21.45)	12 (17.91)	91 (14.96)		
**Lymphatic vessel infiltration**					0.653	0.884
No	418 (88.75)	286 (90.22)	61 (91.04)	43 (88.98)		
Yes	53 (11.25)	31 (9.78)	6 (8.96)	14 (11.02)		

^*^ There was significant difference between patients with normal blood pressure and history of hypertension, the same trend was also found between patients with new diagnosis and history of hypertension (*p* < 0.05).

**Figure 1:**
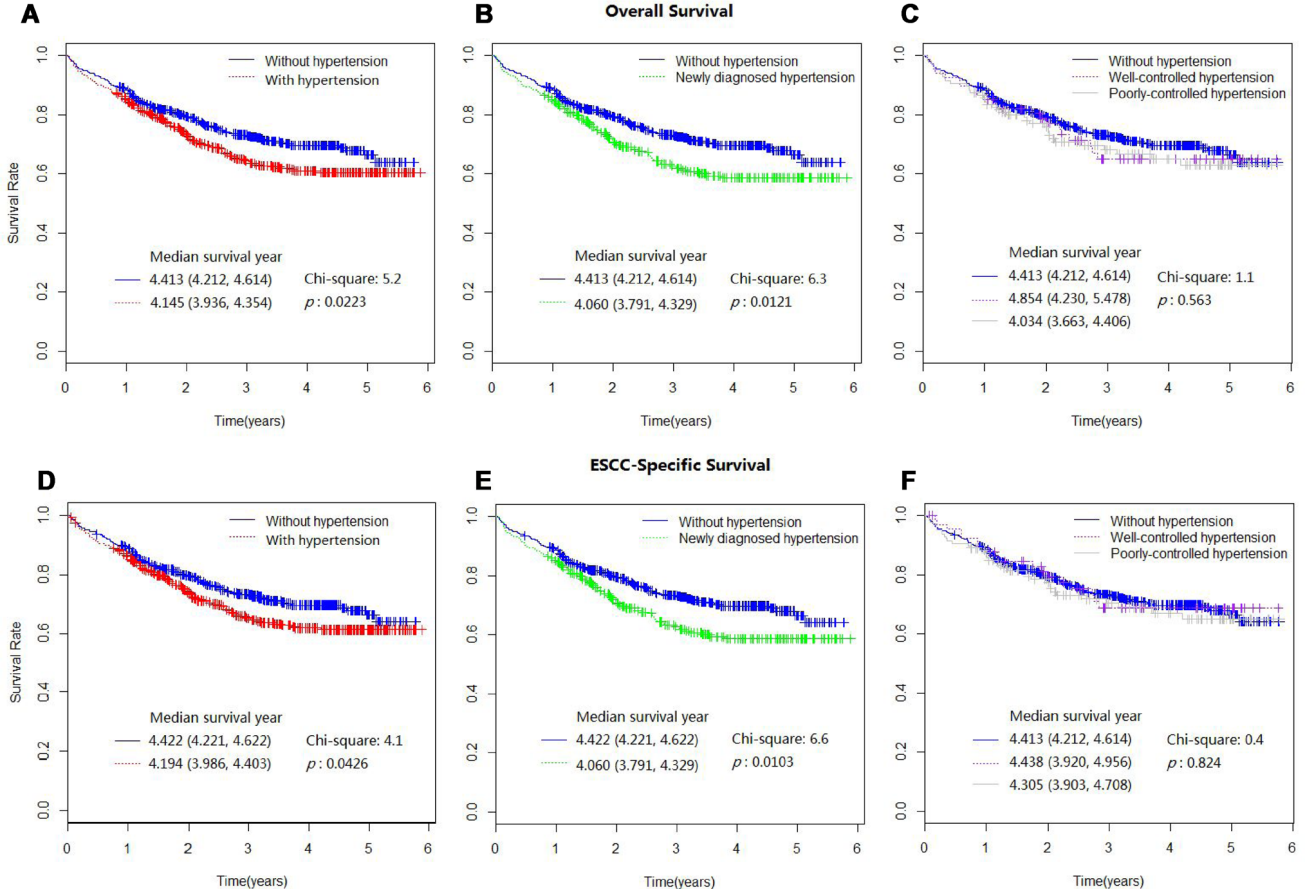
Kaplan-Meier survival curves for overall mortality (**A**–**C**) and ESCC-specific mortality (**D**–**F**), as stratified by different hypertension status.

Kaplan-Meier curves comparing survival outcome stratified by hypertension status were shown in Figure 1. Compared with patients of normal BP, all patients with hypertension had worse overall and ESCC-specific survival outcome (*p* = 0.0223, *p* = 0.0426). Similar association was observed in newly diagnostic hypertension (*p* = 0.0121, *p* = 0.0103). While no significant difference was found among patients with well-controlled, poorly-controlled hypertension and normal BP (*p* = 0.563, *p* = 0.824).

### Comorbidities associated with ESCC survival outcomes

The associations of ESCC prognosis and comorbidities including hypertension were available in Table 2. In univariate Cox proportional hazards regression model, hypertension (HR: 1.305, 95% CI: 1.038, 1.641; HR: 1.270, 95% CI: 1.007, 1.600), cerebral-vascular disease (HR: 1.794, 95% CI: 1.223, 2.633; HR: 1.704, 95% CI: 1.146, 2.534) and chronic obstructive pulmonary disease (COPD) (HR: 1.978, 95% CI: 1.451, 2.696; HR: 1.937, 95% CI: 1.413, 2.656) were associated with higher risk of overall and ESCC-specific mortality.

**Table 2:** Risk for mortality after surgery for ESCC among patients with and without hypertension and other comorbidities

Comorbidities	Overall		HR (95% CI)	*p*	Disease-specific		HR (95% CI)	*p*
	Alive	Dead			Alive	Dead		
**Hypertension**								
No	343 (50.22)	128 (42.81)	1.00 (ref)		344 (49.86)	127 (43.49)	1.00 (ref)	
Yes	340 (49.78)	171 (57.19)	1.305 (1.038, 1.641)	0.0228	346 (50.14)	165 (56.51)	1.270 (1.007, 1.600)	0.0433
**Heart disease**								
No	644 (94.29)	290 (96.99)	1.00 (ref)		650 (94.20)	284 (97.26)	1.00 (ref)	
Yes	39 (5.71)	9 (3.01)	0.604 (0.311, 1.172)	0.1359	40 (5.80)	8 (2.74)	0.548 (0.271, 1.107)	0.5480
**Cerebral-vascular disease**								
No	643 (94.14)	270 (90.30)	1.00 (ref)		648 (93.91)	265 (90.75)	1.00 (ref)	
Yes	40 (5.86)	29 (9.70)	1.794 (1.223, 2.633)	0.0028	42 (6.09)	27 (9.25)	1.704 (1.146, 2.534)	0.0084
**COPD**								
No	624 (91.36)	251 (83.95)	1.00 (ref)		629 (91.16)	246 (84.25)	1.00 (ref)	
Yes	59 (8.64)	48 (16.05)	1.978 (1.451, 2.696)	<0.0001	61 (8.84)	46 (15.75)	1.937 (1.413, 2.656)	<0.0001
**CKD**								
No	671 (98.24)	293 (97.99)	1.00 (ref)		678 (98.26)	286 (97.95)	1.00 (ref)	
Yes	12 (1.76)	6 (2.01)	1.356 (0.604, 3.046)	0.4603	12 (1.74)	6 (2.05)	1.395 (0.621, 3.132)	0.4204
**Diabetes**								
No	648 (94.88)	278 (92.98)	1.00 (ref)		653 (94.64)	273 (93.49)	1.00 (ref)	
Yes	35 (5.12)	21 (7.02)	1.327 (0.852, 2.068)	0.2110	37 (5.36)	19 (6.51)	1.222 (0.768, 1.946)	0.3977
**Endocrine disease**								
No	657 (96.19)	285 (95.32)	1.00 (ref)		661 (95.80)	282 (96.58)	1.00 (ref)	
Yes	26 (3.81)	14 (4.68)	1.173 (0.686, 2.007)	0.5596	29 (4.20)	10 (3.42)	1.203 (0.703, 2.059)	0.4995
**Gastrointestinal disease**								
No	654 (95.75)	289 (96.66)	1.00 (ref)		661 (95.80)	282 (96.58)	1.00 (ref)	
Yes	29 (4.25)	10 (3.34)	0.951 (0.506, 1.787)	0.8763	29 (4.20)	10 (3.42)	0.976 (0.519, 1.834)	0.9390
**Anemia**								
No	682 (99.85)	298 (99.67)	1.00 (ref)		689 (99.86)	291 (99.66)	1.00 (ref)	
Yes	1 (0.15)	1 (0.33)	2.540 (0.356, 18.10)	0.3523	1 (0.14)	1 (0.34)	2.614 (0.367,18.629)	0.3376
**Marasmus**								
No	635 (92.97)	284 (94.98)	1.00 (ref)		641 (92.90)	278 (95.21)	1.00 (ref)	
Yes	48 (7.03)	15 (5.02)	0.754 (0.449, 1.268)	0.2876	49 (7.10)	14 (4.79)	0.719 (0.421, 1.230)	0.2290

COPD: chronic obstructive pulmonary disease; CKD: chronic kidney disease.

### Multivariate-adjusted hazard ratios of hypertension for survival outcome

**Figure 2:**
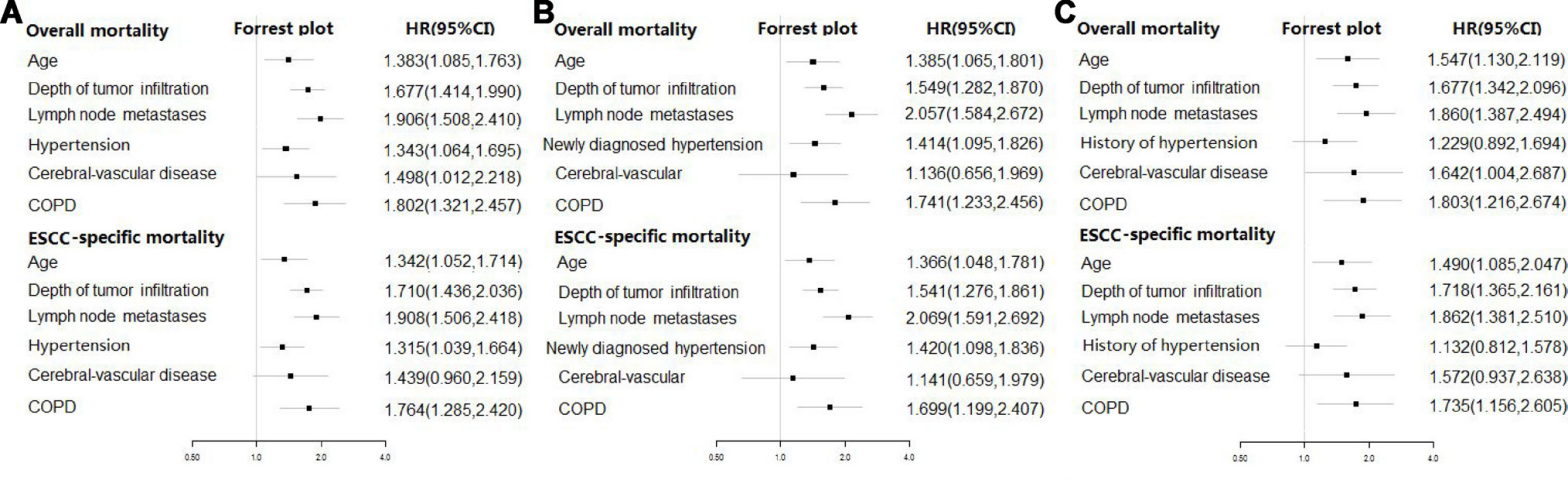
Forest plot of multivariate-adjusted hazard ratios (HRs) of hypertension for overall and ESCC-specific survival outcome (**A**) hypertension was grouped into patients with hypertension and normal BP. (**B**) hypertension was divided into newly diagnosed hypertension and normal BP. (**C**) hypertension was grouped into patients with history of hypertension and normal BP.

We further put patient baseline characteristics, comorbidities and different hypertension status into the multiple Cox proportional hazard regression model. By stepwise variables selecting, the final models expressed as forest plots with HRs for overall and ESCC-specific mortality were shown in Figure 2. After adjustment in multivariate model, older patients (HR: 1.383, 95% CI: 1.085, 1.763; HR:1.342, 95% CI: 1.052, 1.714), deeper of tumor infiltration (HR: 1.677, 95% CI: 1.414, 1.990; HR:1.710, 95% CI: 1.436, 2.036), lymph node metastases (HR: 1.906, 95% CI: 1.508, 2.410; HR:1.908, 95% CI: 1.506, 2.418), hypertension (HR: 1.343, 95% CI: 1.064, 1.695; HR: 1.315, 95% CI: 1.039, 1.664) and COPD (HR: 1.802, 95% CI: 1.321, 2.457; HR:1.764, 95% CI: 1.285, 2.420) were observed as increased risk for overall and ESCC-specific mortality. Patients with cerebral-vascular comorbidity was positively associated with only overall mortality (HR: 1.498, 95% CI: 1.012, 2.218). The similar results were observed that older age (HR: 1.385, 95% CI: 1.065, 1.801; HR: 1.366, 95% CI: 1.048, 1.781), deeper of tumor infiltration (HR: 1.549, 95% CI: 1.282, 1.870; HR: 1.541, 95% CI: 1.276, 1.861), lymph node metastases (HR: 2.057, 95% CI: 1.584, 2.672; HR: 2.069, 95% CI: 1.591, 2.692), newly diagnosed hypertension (HR: 1.414, 95% CI: 1.095, 1.826; HR: 1.420, 95% CI: 1.098, 1.836) and COPD (HR: 1.741, 95% CI: 1.233, 2.456; HR: 1.699, 95% CI: 1.199, 2.407) were associated with increased risk of overall and ESCC-specific mortality in model where hypertension was divided into newly diagnosed and normal BP. However, when the hypertension was grouped into history of hypertension and normal BP, no association was found between history of hypertension and overall or ESCC-specific survival outcomes (HR:1.229, 95% CI: 0.892, 1.694; HR: 1.132, 95% CI: 0.812, 1.578).

## DISCUSSION

The aim of our study was to investigate the relationship between hypertension and prognosis of patients with ESCC after surgical resection. To our knowledge, there was no previous studies conducted the relationships between ESCC prognosis and hypertension. The main finding of our study was that hypertension before esophagectomy shows an unfavorable prognosis for ESCC patients.

Hypertension, a chronic systemic disorder, has emerged as a pivotal factor of cardiovascular disease. It is notable that several studies have reported preoperative hypertension as a significant etiologic factor for the prognosis of cancers. Among patients with nasopharyngeal carcinoma, preoperative hypertension is an independent factor and results in poorer survival outcomes [24]. The similar trend has been found in patients with ovarian cancer [25]. In addition, hypertension alone reveals the increased risk of prior prognosis in bladder cancer after Bacilllus Calmette Guerin treatment [26]. It is also considered to be related to inferior prognosis of prostate cancer [27]. When age, race, tumor characteristics and breast cancer treatment were controlled, the presence of hypertension was associated with poorer breast cancer outcome [21].

If hypertension is truly causally associated with cancers, the biologic mechanisms linking hypertension and cancer should be elucidated further. However, the mechanisms are still not clear. In previous studies, the following possible mechanisms have been pointed out, but we have not found the subsequent studies focused on further discussion and confirmation. Based on the prior studies, increased expression of cytosolic calcium and inositol triphosphate involved in the pathogenesis of hypertension have been hypothesized to be involved in the early events of cell proliferation that may activate the endogenous oncogenes [28]. In addition, aberrant carcinogen has been found to bind to deoxyribonucleic acid in lymphocytes of hypertensive patients [29].

In recent studies, chronic hypoxia has been delineated as one possible mechanism that might be partly responsible for the increased cancer mortality in patients with hypertension [30]. Hypertension caused microvascular alterations in function and structure. Microvascular alterations, amplifying the haemodynamic load within microvascular network and inducing vasoconstriction within the microcirculation, could further lead to chronic endothelial injury and oxidative stress and promote tissue hypoxia [31]. Such adverse oxygenation status can promote tumor progression and contribute to an unfavorable prognosis for patient outcome [32].

Recently, as a meta-analysis having proved that the vascular endothelial growth factor (VEGF) overexpression associated with worse survival outcome in patients with ESCC, the connections of VEGF and hypertension could explain the major reason why ESCC patients with hypertension had the inferior prognosis [33]. VEGF has been considered as one of the most comprehensively prognostic biomarkers in a wide variety of tumors, including ESCC [34]. Thus far, it has been identified as one of the most potent stimulators involved in tumor-associated angiogenesis [35].

There was inconsistent association between history of hypertension and survival outcomes among cancer patients. In one prospective study, a negative association has been observed between history of hypertension and overall survival among patients with ovarian cancer [36]. Conversely, one retrospective study has failed to find an association between them among patients with ovarian cancer [37]. For endometrial cancer, there is an evidence that reduced mortality was associated with a history of hypertension [38, 39]. The authors of above studies of endometrial cancer hypothesized that antihypertensive treatment might be responsible for the reduced risk of mortality. It is plausible that antihypertensive medications could have a differential effect on a hormonal admixture in the patient's body and may influence the tumor microenvironment differently. However, this association is still lacking in tested biological mechanisms. In our study, we have not found the significant association between history of hypertension and survival outcome. The detailed mechanism should be further examined in the future study.

Through multiple analyses, patients with increased age have worse survival outcomes after esophagectomy. On the one hand, the operation is established in patients diagnosed with resectable tumors, which are in good clinical condition, whereas elder patients have relatively poor clinical condition and high risk of comorbidities [40]. From another point of view, we found that the percentage of hypertension distributed higher in elder patients. Unfortunately, the existing researches failed on considering hypertension as potential factors affecting prognosis among ESCC patients. Except for hypertension, cerebral-vascular disease and COPD were also found to increase the risk of ESCC mortality. Cerebral-vascular disease, a common risk factor of brain cancer prognosis, has been found to associated with lymph node metastasis in breast cancer patients [41]. COPD, an inflammatory respiratory disorder that related to inferior prognosis of lung cancer, has been identified that its pathogenesis is affected by tumor necrosis factor-related apoptosis-inducing ligand (TRAIL), which is known as tumor necrosis factor superfamily member 10 (TNFSF10) [42]. Since there is not existing research about relationships between the above chronic disease and ESCC prognosis, the in-depth study should be further exploring. Besides, we found that Kaplan-Meier curves showed a tendency to be closer after the fifth year of follow-up. This might be the result of the gradually increased impact caused by age and above comorbidities during the long-term follow-up. In addition, patient lost to follow up happening gradually over time might be another reason.

Several limitations should be kept in mind of this study. First, this is a retrospective, observational study at a single institution. Second, we only considered patients with ESCC cause fewer patients diagnosed with esophageal adenocarcinoma or neuroendocrine carcinomas, which limited us to further comparative analyses. Third, we only focused on the preoperative hypertension, the hypertension that may occur during in the follow-up interval has not been detected. Fifth, the findings presented in this study cannot be directly extrapolated to the general populations as only patients who received esophagectomy were eligible for inclusion. Furthermore, the biologic mechanisms linking hypertension and ESCC should be elucidate in the next work.

In conclusion, we revealed the evidence that hypertension was associated with poorer prognosis among ESCC patients. From a public health perspective, these results were important because hypertension was highly prevalent worldwide and its control remained inadequate. Our analysis supported the relevance of public health programs aimed at improving the prognosis of patients with ESCC.

## MATERIALS AND METHODS

### Patients

Between August 2010 and December 2015, a consecutive series of 2040 patients who were diagnosed with EC and underwent esophagectomy were enrolled at Shanxi Provincial Cancer Hospital. Patients included in this study met the following criteria: (i) tumors histopathologically confirmed as ESCC; (ii) underwent curative esophagectomy; (iii) without neoadjuvant chemotherapy or radiotherapy; (iv) no other primary cancer or history of other cancers (v) with pathologically negative resection margin. Finally, a total of 982 patients were recruited in this study. The study was approved by the Ethics Committee of Shanxi Provincial Cancer Hospital, and the written informed consents was obtained from all patients before surgery.

### Follow-up

All patients were routinely followed up every 6 months for 3 years after undergoing curative surgery and annually thereafter. In this study, the follow-up was completed in December 2016. Patients were traced by telephone interviewers and the death information was confirmed by their family and local mortality registration department. Both overall and ESCC-specific survival time were recorded from the date of surgery until the date of death. Patients who were still alive at the last follow-up were treated as censored data with the censored rate of 70.63%.

### Measurement and definition

The general information was collected in a face-to-face interview using anonymous questionnaires by well-trained nurses at the time of enrollment. The questionnaire contains questions on birth, gender, alcohol consumption, current smoking and familial history of chronic diseases. Current alcohol users were defined as consuming alcohol at least once per month in the past year [43]. Current smokers were participants who have smoked at least one cigarette per day during the past month [44]. Information on history of chronic disease, comorbidity and tumor characteristics was obtained from the medical records. Primary tumor, regional lymph nodes and distant metastasis were coded based on the 7th edition of AJCC cancer staging manual [45]. Hypertension was defined as the average SBP ≥ 140 mmHg and/or DBP ≥ 90 mmHg according to the ambulatory BP monitor, as well as having history of hypertension or receiving antihypertension medication treatment [46, 47]. BP were measured by portable BP monitors (HEM-7120, Omron, Liao Ning, China) every 30 min throughout the whole day while patients went about their normal activities and sleeping in the day of being hospitalized [48]. In this study, hypertension was categorised as newly diagnostic hypertension and history of hypertension. Patients with average SBP ≥ 140 mmHg and/or DBP ≥ 90 mmHg for the first time during this hospitalization phrase were defined as newly diagnosed hypertension. The history of hypertension was patients with prior diagnosed hypertension or taking antihypertension medical therapy. Among them, patients with BP measurement lower than 140/90 mmHg were defined as well-controlled hypertension, while BP measurement equal to or higher than 140/90 mmHg were defined as poorly-controlled hypertension.

### Statistical analyses

Statistical analyses were conducted with SAS statistical software (Version 9.3, SAS Institute, Inc., Cary, NC, USA). Categorical variables were described by frequencies and percentages and examined by Chi-square and rank test for unordered and ordinal categorical variables, respectively. Kaplan-Meier method and Log-rank test were used to estimate survival curves and compare cumulative survival differences. We assessed the impact of hypertension on survival outcome using Cox proportional hazards regression model to calculate hazard ratios (HRs) and 95% confidence intervals (CIs). The forest plot was used to reveal the regression results. Significance was two-sided and set at *p* < 0.05.

## Abbreviations

EC: esophageal cancer
ESCC: esophageal squamous cell carcinoma
BP: blood pressure
VEGF: vascular endothelial growth factor
SBP: systolic blood pressure
DBP: diastolic blood pressure
COPD: chorinic obstructive pulmonary disease
CKD: chronic kidney disease
AJCC: American Joint Committee on Cancer
